# Gabapentin dose and the 30-day risk of altered mental status in older adults: A retrospective population-based study

**DOI:** 10.1371/journal.pone.0193134

**Published:** 2018-03-14

**Authors:** Jamie L. Fleet, Stephanie N. Dixon, Paul John Kuwornu, Varun K. Dev, Manuel Montero-Odasso, Jorge Burneo, Amit X. Garg

**Affiliations:** 1 Department of Physical Medicine and Rehabilitation, McMaster University, Hamilton, Ontario, Canada; 2 Institute for Clinical Evaluative Sciences, London, Ontario, Canada; 3 Department of Epidemiology & Biostatistics, Western University, London, Ontario, Canada; 4 Division of Nephrology, Department of Medicine, Sunnybrook Hospital, Toronto, Ontario, Canada; 5 Division of Geriatrics, Department of Medicine, Western University, London, Ontario, Canada; 6 Department of Clinical Neurological Sciences, Western University, London, Ontario, Canada; 7 Division of Nephrology, Department of Medicine, Western University, London, Ontario, Canada; Kaohsiung Medical University Hospital, TAIWAN

## Abstract

Gabapentin is an effective treatment for chronic neuropathic pain but may cause dizziness, drowsiness, and confusion in some older adults. The goal of this study was to assess the association between gabapentin dosing and adverse outcomes by obtaining estimates of the 30-day risk of hospitalization with altered mental status and mortality in older adults (mean age 76 years) in Ontario, Canada initiated on high dose (>600 mg/day; n = 34,159) compared to low dose (≤600 mg/day; n = 76,025) oral gabapentin in routine outpatient care. A population-based, retrospective cohort study assessing new gabapentin use between 2002 to 2014 was conducted. The primary outcome was 30-day hospitalization with an urgent head computed tomography (CT) scan in the absence of evidence of stroke (a proxy for altered mental status). The secondary outcome was 30-day all-cause mortality. The baseline characteristics measured in the two dose groups were similar. Initiation of a high versus low dose of gabapentin was associated with a higher risk of hospitalization with head CT scan (1.27% vs. 1.06%, absolute risk difference 0.21%, adjusted relative risk 1.29 [95% CI 1.14 to 1.46], number needed to treat 477) but not a statistically significant higher risk of mortality (1.25% vs. 1.16%, absolute risk difference of 0.09%, adjusted relative risk of 1.01 [95% CI 0.89 to 1.14]). Overall, the risk of being hospitalized with altered mental status after initiating gabapentin remains low, but may be reduced through the judicious use of gabapentin, use of the lowest dose to control pain, and vigilance for early signs of altered mental status.

## Introduction

Most of the data to guide gabapentin use and dosing in older adults is from pharmacokinetic studies or case reports[1,2] While gabapentin is approved to prevent seizures, most patients take gabapentin for reasons of neuropathic pain (71%) or psychiatric disorders, with bipolar being most common (15%), with an average dose of 975 mg per day, ranging from 100 to 4800 mg per day[3]. There are some inconsistent recommendations for an appropriate standard dose of gabapentin in prescribing references (Table 1), where the recommended dose varies by medical indication[4–6], and in practise is often titrated up to maximum tolerable dose[2]. It may be reasonable to start older adults on a low dose of gabapentin, which can be effective to treat pain while exposing patients to a lower risk of adverse mental status side effects of gabapentin (dizziness, drowsiness and confusion)[7]. For example, in several studies titrating gabapentin to a maximum possible dose (generally 3,600mg per day) was not necessarily more effective than a low dose[7,8]. In a randomized placebo controlled trial, clinically relevant pain relief was achieved at doses as low as 900mg daily in 43% of patients (mean age 62 years)[9]. It may be particularly important to dose-reduce gabapentin in the presence of chronic kidney disease, a condition common in older adults which results in higher than average plasma concentrations of gabapentin as this drug is eliminated almost entirely by the kidney[10,11]. However, in practice older adults are frequently not initiated on low doses of gabapentin[2]. We conducted this population-based cohort study to understand the risk of acute altered mental status and mortality within 30 days of initiating a high versus low dose of gabapentin in older adults, a segment of the population at higher risk of adverse drug events. We also considered whether any observed effects were altered by the presence of chronic kidney disease.

**Table 1:** Gabapentin dosing recommendations in popular drug prescribing references.

High Dose*	Low Dose*	Package Insert(6)	UpToDate(5)	Medscape(4)
>600 mg/day	≤600 mg/day	Epilepsy: 900–1800 mg/day	Epilepsy: Initial dose of 900 mg/day though doses of 2,400 mg/day have been tolerated	Epilepsy: 900 mg/day (may increase to max of 1800mg/day)
		Postherpatic neuralgia: 300mg on day 1; 600mg on day 2; 900 mg on day 3 and later	Pain: 300 mg on day 1; 600 mg on day 2; 900 mg on day 3 and later to a max of 1,800 mg (as higher doses don't show greater benefit)	Postherpatic neuralgia:300mg on day 1; 600mg on day 2; 900 mg on day 3 and later
		Geriatric: Because elderly patients are more likely to have decreased renal function, care should be taken in dose selection, and dose should be adjusted based on creatinine clearance values in these patients	Geriatric: Same as adult dosing	Geriatric: can titrate gradually to max of 1800mg/day
		Renal impairment (CrCl in mL/min): 30–59 = 400-1400mg/day 16–29 = 200–700mg/day ≤15 = 100–300 mg/day	Renal impairment: CrCl in mL/min): 30–59 = 400–1400 mg/day 16–29 = 200–700mg/day ≤15 = 100–300 mg/day	Renal impairment (CrCl in mL/min): 30–60 = 400-1400mg/day 15–29 = 200-700mg/day <15 = 100-300mg/day

* Low dose and high dose were defined a priori based on a variety of factors including assessment of average daily dosing in our jurisdiction.

## Methods

### Study design

We conducted a retrospective population-based cohort study using linked administrative databases. The province of Ontario, Canada has approximately 14 million residents, 16% (2.3 million) of whom are 65 years of age or older[12]. These older residents have universal prescription drug coverage. This study was approved by the institutional review board at Sunnybrook Health Sciences Centre, Toronto, Canada. The reporting of this study followed guidelines for observational studies (S1 Table)[13].

### Data sources

We ascertained outcomes as well as the presence of relevant comorbidities for exclusions and baseline characteristics using records from population databases linked using unique encoded identifiers and analyzed at the Institute for Clinical Evaluative Sciences (ICES). The Ontario Drug Benefit Plan (ODB) database contains records of prescriptions from outpatient pharmacies. The dispensing of medications for patients aged 65 and older is accurately recorded in this database with an error rate of less than 1%[14]. The Canadian Institute for Health Information (CIHI) National Ambulatory Care Reporting System (NACRS) contains ambulatory care information on emergency room visits, outpatient procedures, and day surgeries. The CIHI Discharge Abstract Database (CIHI-DAD) reports inpatient procedures, diagnoses, and discharge summaries for all patients hospitalized in Ontario. The Ontario Health Insurance Plan (OHIP) database contains all physician and other specific health care provider claims for medical services covered under the provincial health insurance plan. The Ontario Mental Health Reporting System (OMHRS) contains information on mental health admissions in Ontario, as well as psychiatric diagnoses and substance use. The Registered Persons Database (RPDB) contains demographic information, such as birth date and sex, for all permanent Ontario residents. The ICES Physician Database (IPDB), a dataset specific to ICES, provides information on physicians’ date of birth, sex, year of graduation, specialty, and setting of practice. Finally, in a subpopulation, we used linked laboratory datasets with serum creatinine information from Cerner and Gamma-Dynacare.

### Patients

Older adults included in our study had at least one new outpatient oral prescription for gabapentin lasting at least seven days between April 1^st^ 2002 and December 31^st^ 2014. The date the gabapentin was dispensed served as the cohort entry date (also referred to as the index date). We excluded the following older adults from analysis: (1) those in their first year of eligibility for prescription drug coverage (aged 65 years) to avoid incomplete medication records; (2) those with a prescription for our study drug (gabapentin) or non-study drug (pregabalin) in the 180 days prior to the index date, to restrict the analysis to new use of gabapentin; (3) those living in a long term care institution as such patients frequently have altered mental status making such events difficult to attribute to medication use; (4) those with end-stage renal disease (defined as chronic dialysis or renal transplant), as gabapentin is readily dialyzable, which alters prescribing recommendations[15,16]; (5) those who were discharged from hospital in the 2 days prior to their index date, or if the index date fell between an admission and discharge, in order to ensure gabapentin prescriptions were not a continuation from a hospital treatment; and (6) those with a prescription for gabapentin greater than 4000 mg per day as this was likely an error. A patient could enter the cohort only once.

In subgroup analyses, we identified patients with chronic kidney disease, which could be defined either with databases codes, or in a subpopulation with laboratory values. In Ontario, the validated algorithm of database codes for chronic kidney disease identifies older adults with a median estimated glomerular filtration rate [eGFR] of 38 mL/ min per 1.73 m^2^ [interquartile range (IQR), 27–52], whereas its absence identifies those with a median eGFR of 69 mL/min per 1.73 m^2^ [IQR, 56–82])[17]. In a subpopulation of patients with available baseline serum creatinine data from outpatient laboratories, we defined chronic kidney disease as an estimated glomerular filtration rate (eGFR) < 45 mL/min per 1.73 m^2^ (based on the most recent serum creatinine prior to the gabapentin prescription). This threshold was chosen *a priori* for reasons of feasibility (choosing a lower eGFR threshold of < 30 mL/min per 1.73 m^2^ would mean too few patients for analysis in our dataset) and for reasons of biology (a higher eGFR threshold of 45 to 60 mL/min per 1.73 m^2^ may not identify substantial chronic kidney disease in the elderly). In truth, the best equation to estimate kidney function for the purposes of drug adjustment is controversial–the United States Kidney Disease Education program indicates that equations which express results in mL/min per 1.73 m^2^ or mL/min are both appropriate for this purpose. In this study, we estimated GFR using the Chronic Kidney Disease–Epi equation (CKD-EPI), which when less than 45 mL/min per 1.73 m^2^ would also generally identify a patient with a Cockcroft-Gault result less than 45 mL/min (at this level of kidney function, agreement between both equations is good, although the CKD-Epi equation generally yields a higher estimate of eGFR in older adults).

### Gabapentin dose

The main comparison in this study was between two groups of patients prescribed either a high or low dose of gabapentin. In this study we assessed the average daily dose of gabapentin from the first (initial) prescription, meaning it was the average daily dose of all doses received for the duration of the initial prescription. In this study we classified daily doses of 600 mg or less as “low dose” and doses above 600 mg as “high dose” prior to any outcome analyses. This was justified for several reasons. In a healthy adult being treated for pain and/or postherpetic neuralgia, it is recommended gabapentin be started at 300mg per day on the first day, 600mg the second day and 900mg the third day and beyond[18]. In older adults it is recommended to start at a lower dose and titrate up for a desired effect as necessary, although guidance rarely goes beyond that. Many older patients have reduced kidney function and an initial dose ranging from 200 to 1400 mg/day in patients with a creatinine clearance of 20–59 mL/min is recommended. Finally, we assessed the distribution of dosages in our region used in common practice *a priori*; a 600 mg cut-off point provided a reasonable number of persons in each of our two dosing groups.

### Outcomes

Gabapentin-related altered mental status changes, when they occur, frequently do so in the first few weeks of drug initiation[1,19]. For this reason, we followed all individuals for 30 days after they initiated gabapentin for two pre-specified outcomes. The primary outcome was hospitalization with an urgent head computed tomography (CT) scan in the absence of a diagnosis of stroke, which in our data sources was used as a proxy for the development of new, significant altered mental status. In the clinical setting of acute altered mental status presenting to hospital where no alternative explanation is apparent on first evaluation (as in the case of most patients with drug-induced altered mental status) the general standard of care in our jurisdiction is to perform an urgent head CT scan. Unlike many hospital diagnostic codes for mental status changes, such as delirium which have very poor accuracy, completion of a head CT scan is very well coded in our data sources, similar to other fee-for-service codes associated with physician reimbursement[20]. To focus on acute altered mental status, we limited this outcome to only head CT scans performed during the first five days of the hospital admission or in the emergency department preceding the admission, and excluded those where stroke was listed as the most responsible diagnosis. The secondary outcome of this study was all-cause mortality, which is well coded in our data sources (sensitivity of 94% and positive predictive value of 100%)[21].

In addition to our two primary clinical outcomes we also compared our two gabapentin dose groups on estimated 30-day healthcare sector specific costs, such as emergency department visits and inpatient hospital admissions, as well as total healthcare costs in the 30-day follow-up. The method of assessing healthcare cost in our data sources is fully described elsewhere[22]. Total healthcare cost was the sum of costs of physician visits, long term care, complex continuing care, home care, emergency department visits, inpatient hospital admissions, rehabilitation, prescription drugs, and same day surgeries. All costs are expressed in 2014 Canadian dollars.

### Statistical analysis

We compared baseline characteristics between those prescribed a high versus low daily dose of gabapentin using standardized differences. This metric describes differences between group means relative to the pooled standard deviation and is considered a meaningful difference if greater than 10%[23]. We expressed the risk of developing an outcome in both relative and absolute terms. Absolute risk was also expressed as the number needed to harm (NNH) (1 / absolute risk difference). This measure indicates how many patients need to receive a high dose of gabapentin to cause harm to one patient who otherwise would not have been harmed if all patients received a low dose gabapentin (a lower number indicating greater harm).

All analyses were performed using SAS version 9.4. We conducted multivariable logistic regression analyses to estimate odds ratios and 95% confidence intervals. The odds ratio approximates the relative risk when the event is rare (as found for our condition). We adjusted for eight potential pre-specified characteristics: age, sex, year of cohort entry, Charlson comorbidity score (a composite of comorbidities predicting risk of one year mortality)[24], baseline evidence of dementia and trigeminal neuralgia, as well as baseline use of antiepileptic and narcotic prescriptions. We evaluated the association between gabapentin dose (high vs. low) and outcomes in the pre-specified two subgroups of chronic kidney disease (as assessed by administrative codes [listed in S2 Table], or by eGFR in a subpopulation with available laboratory values). Additional analyses were completed after knowledge of the primary results (see Results section). In all outcome analyses we interpreted 2-tailed *P* values lower than 0.05 as statistically significant.

We compared the adjusted mean costs between patients who were prescribed high and low daily dose of gabapentin. Mean costs were adjusted for the same eight characteristics as above; using a general linear model assuming a normal distribution for costs and identity link function. An additional analysis was done to assess the avoidable healthcare costs associated with preventing hospitalizations for urgent head CT scans due to initiating a high dose of gabapentin. We estimated the attributable risk fraction using the adjusted relative risk of hospitalization for head CT scans due to initiating a high dose of gabapentin. Avoidable healthcare costs were calculated based on the estimated attributable risk fraction in our cohort. We also included estimates of avoidable healthcare costs for varying levels of prevalence of initiating a high dose of gabapentin.

## Results

We identified 148,769 patients who initiated gabapentin during our study period. Cohort selection is presented in S1 Fig. After applying our exclusions, 110,184 unique patients remained who received a high dose (n = 34,159) or low dose (n = 76,025) of gabapentin. Most prescriptions were written by a primary care physician (68.7%). Hospitalizations with altered mental status took place across 121 different hospitals in Ontario.

Baseline characteristics of the two dosing groups were similar (select baseline characteristics presented in Table 2, with full results reported in S3 Table). Men were more likely to receive a high dose (42.9% vs. 35.7%), as were younger patients (median age 73 vs. 76 years). Patients that entered the cohort in the last three years of accrual were more likely to receive a low dose (60.8% vs. 44.2%).

**Table 2:** Baseline characteristics.

	High Dose^¶^ n = 34,159	Low Dose^£^ n = 76,025	Standardized Difference (%)*
Age, mean (SD)	74.4 (6.5)	76.3 (7.2)	28%
Women	19,508 (57.1)	48,906 (64.3)	15%
Year of cohort entry			
2002–2005	4,067 (11.9)	4,272 (5.6)	22%
2006–2009	6,187 (18.1)	8,219 (10.8)	21%
2010–2013	19,563 (57.3)	48,743 (64.1)	14%
2014	4,342 (12.7)	14,791 (19.5)	19%
Rural	5,418 (15.9)	10,464 (13.8)	6%
Income quintile			
Missing	247 (0.3)	133 (0.4)	2%
1 (lowest)	7,049 (20.6)	16,120 (21.2)	1%
2	6,997 (20.5)	16,009 (21.1)	1%
3	6,655 (19.5)	15,179 (20.0)	1%
4	6,743 (19.7)	14,621 (19.2)	1%
5 (highest)	6,582 (19.3)	13,849 (18.2)	3%
*Comorbidities in 5 years prior*			
Alzheimer’s disease	62 (0.2)	238 (0.3)	2%
Atrial fibrillation/flutter	2,414 (7.1)	5,969 (7.9)	3%
Cardiovascular disease^#^	10,056 (29.4)	22,693 (29.8)	1%
Chronic liver disease	1,599 (4.7)	3,328 (4.4)	1%
Chronic obstructive pulmonary disease	2,105 (6.2)	4,460 (5.9)	1%
Dementia	3,153 (9.2)	8,866 (11.7)	8%
Diabetes Mellitus	8,650 (25.3)	20,008 (26.3)	2%
Congestive heart failure	4,663 (13.7)	11,683 (15.4)	5%
Migraine	2,181 (6.4)	4,370 (5.7)	3%
Neuropathic Pain	1,778 (5.2)	2,675 (3.5)	8%
Peripheral vascular disease	1,068 (3.1)	1,871 (2.5)	4%
Seizure disorder	528 (1.5)	728 (1.0)	5%
Sepsis	576 (1.7)	1,109 (1.5)	2%
Stroke	1,227 (3.6)	2,602 (3.4)	1%
Trigeminal Neuralgia	2,249 (6.6)	3,205 (4.2)	11%
*Tests/Procedures in the one year prior*			
Cardiac catheterization	771 (2.3)	1,561 (2.1)	1%
CT head	5,628 (16.5)	12,067 (15.9)	2%
Electroencephalogram	583 (1.7)	871 (1.1)	5%
Prescribing Physician Characteristics^**^			
Time since graduation, mean (SD)	26.2 (11.7)	25.7 (11.7)	4%
*Specialty*			
GP	22,346 (65.4)	53,403 (70.2)	10%
Anesthesiologist	441 (1.3)	1,102 (1.4)	1%
Nephrology	25 (0.1)	336 (0.4)	6%
Cardiology	79 (0.2)	183 (0.2)	0%
Neurology	2,510 (7.3)	3,184 (4.2)	13%
Physical Medicine and Rehab	400 (1.2)	805 (1.1)	1%
Missing	9,145 (12.0)	5,037 (14.7)	8%
Other	3,321 (9.7)	7,867 (10.3)	2%
*Medications in 180 days prior*			
ACE-inhibitors	10,712 (31.4)	22,834 (30.0)	3%
ARBs	5,886 (17.2)	15,186 (20.0)	7%
Anti-depressants	11,984 (35.1)	24,114 (31.7)	7%
Anti-epileptics	2,418 (7.1)	3,188 (4.2)	13%
Antipsychotics	1,485 (4.3)	3,839 (5.0)	3%
Beta-blockers	9,831 (28.8)	23,096 (30.4)	4%
Calcium channel blockers	10,102 (29.6)	24,239 (31.9)	5%
Diuretics	11,172 (32.7)	25,337 (33.3)	1%
Histamine-2 receptor antagonist	2,546 (7.5)	5,306 (7.0)	2%
Statins	16,982 (49.7)	39,603 (52.1)	5%
Benzodiazepines	7,732 (22.6)	16,783 (22.1)	1%
Cholinesterase inhibitors	709 (2.1)	2,243 (3.0)	6%
Migraine therapies	15 (0.03)	20 (0.04)	6%
Narcotics and Narcotic Antagonists	18,516 (54.2)	36,136 (47.5)	13%

Abbreviations: SD, standard deviation; CT, computed tomography; GP, general practitioner; CMG, Canadian Medical Graduate; ACE, angiotensin converting enzyme; ARB, angiotensin receptor blocker; NSAIDs, nonsteroidal anti-inflammatory drug; ASA, acetylsalicylic acid

^¶^High dose of gabapentin defined as >600 mg/day

^£^Low dose of gabapentin defined as ≤600 mg/day

*Standardized differences are less sensitive to sample size than traditional hypothesis tests. They provide a measure of the difference between groups divided by the pooled standard deviation; a value greater than 10% is interpreted as a meaningful difference between the groups

#Coronary artery disease incorporates coronary artery revascularization as well as myocardial infarction but does not include angina

**Physicians may be presented more than once if they had written prescriptions for more than 1 patient present in our cohort.

There were 15,283 unique physicians that prescribed an index prescription, with 2,550 of those prescribing only high doses, 5,847 prescribing only low doses, and 6,886 prescribing both doses. The mean number of prescriptions written per physician was 7 (standard deviation 14).

### 30-day hospitalization with altered mental status

These results are presented in Table 3. Initiating a high versus low dose of gabapentin was associated with a higher risk of hospitalization with altered mental status (434 patients of 34,159 taking a high dose [1.27%] vs. 809 of 76,025 taking a low dose [1.06%]; adjusted relative risk 1.29 [95% CI 1.14 to 1.46], p-value <0.0001). The absolute risk difference was 0.21% (95% CI 0.07 to 0.35). The number needed to harm was 477 (95% CI 286 to 1429).

**Table 3:** 30-day primary and secondary outcomes.

	Number of events, n (%)		Relative Risk (95% CI)		Absolute Risk Difference, % (95% CI)	Number Needed to Harm^#^ (95% CI)
	High Dose^¶^ N = 34,159	Low Dose^£^ N = 76,025	Unadjusted	Adjusted^¥^		
Hospitalization with altered mental status*	434 (1.27)	809 (1.06)	1.20 (1.06–1.35)	1.29 (1.14–1.46)	0.21 (0.07–0.35)	477 (286–-1429)
**All-cause mortality**	426 (1.25)	883 (1.16)	1.07 (0.96–1.21)	1.01 (0.89–1.14)	0.09 (0.05–0.23)	not reported^$^

Abbreviations: CI, confidence interval.

^¶^High dose of gabapentin defined as >600 mg/day.

^£^Low dose of gabapentin defined as ≤600 mg/day.

^¥^Adjusted for 8 covariates (see Methods).

* Altered mental status as defined by receipt of urgent head CT scan in the absence of diagnosis of stroke within the first 5 days of hospital admission as diagnosed by hospital administrative codes.

#Number needed to harm rounded up to nearest whole number. It does not apply causality, as all results are associations but is there for ease of interpretation.

$Number needed to harm was not reported for all-cause mortality as there was no statistical increase in risk.

Patients prescribed the low gabapentin dose served as the referent group.

Results from subgroup analyses by baseline chronic kidney disease status are presented in Table 4. The risk of hospitalization with altered mental status with a high versus low dose of gabapentin was higher in patients with chronic kidney disease than in patients without chronic kidney disease. This appeared true when chronic kidney disease was assessed by the presence of database codes (p-value for interaction 0.017). However, CKD defined using laboratory values was not technically statistically significant at the 0.05 level of significance (p-value for interaction p-value 0.054).

**Table 4:** Subgroup analysis for primary outcome of hospitalization with altered mental status in 30 day follow up.

CKD Status	Dose^$^	Number of patients	Number of events^⏡^, n (%)	Relative Risk (95% CI)		Adjusted p- value	Adjusted interaction p-value	Absolute risk difference % (95% CI)	Number needed to harm
				Unadjusted	Adjusted				
Chronic kidney disease^*^	Low Dose	8,345	158 (1.89)	1.0 (ref)	1.0 (ref)	0.0003	0.017	0.98 (0.35–1.70)	102
	High Dose	2,955	85 (2.88)	1.53 (1.18–2.00)	1.68 (1.27–2.22)				
No chronic kidney disease^*^	Low Dose	67,680	651 (0.96)	1.0 (ref)	1.0 (ref)	0.003		0.16 (0.02–0.30)	639
	High Dose	31,204	349 (1.12)	1.16 (1.02–1.33)	1.22 (1.07–1.40)				
**eGFR <45**^**#**^	Low Dose	2,729	51 (1.87)	1.0 (ref)	1.0 (ref)	0.04	0.054	1.20 (0.07–2.66)	84
	High Dose	848	26 (3.07)	1.66 (1.03–2.68)	1.67 (1.02–2.73)				
**eGFR ≥45**^**#**^	Low Dose	15,454	152 (0.98)	1.0 (ref)	1.0 (ref)	0.62		0.02 (-0.33–0.25)	4167
	High Dose	6,653	67 (1.01)	1.02 (0.77–1.37)	1.08 (0.80–1.45)				

Abbreviations: CKD, chronic kidney disease; eGFR, estimated glomerular filtration rate

$ Low dose defined as ≤600mg per day of gabapentin. High dose defined as >600mg per day of gabapentin.

⏡ Events are defined by receipt of urgent head CT scan in the absence of diagnosis of stroke within the first 5 days of hospital admission as diagnosed by hospital administrative codes.

* Chronic kidney disease as defined by presence of at least one administrative database code from a previously validated algorithm. Listing of codes is presented in Appendix B

# eGFR is based on subgroup analysis of patients that have a recent serum creatinine laboratory value from Gamma-Dynacare or Cerner.

Patients prescribed the low gabapentin dose served as the referent group.

### 30-day all-cause mortality

Initiating a high versus low dose of gabapentin was not associated with a significantly higher risk of death (426 patients of 34,159 taking a high dose [1.25%] vs. 883 of 76,025 taking a low dose [1.16%]; adjusted relative risk 1.01 [95% CI 0.89 to 1.14]. There was also a lack of association between gabapentin dose and mortality in patients with and without chronic kidney disease (p-value for interaction 0.97 using database codes, and 0.35 for laboratory values; S4 Table).

### Additional analyses

The primary associations were also robust in additional analyses. First, we redefined our primary outcome as evidence of urgent neuroimaging within 2 days (vs. 5 days) of a hospital admission; with the rationale that neuroimaging done earlier in the hospital stay was more likely related to the reason for hospital presentation. An outpatient high (vs. low) dose of gabapentin remained associated with a higher risk of hospitalization with an urgent CT head when defined this way (adjusted odds ratio 1.29, 95% CI 1.34 to 1.46; S5 Table). Second, to confirm the two groups of patients initially categorized by gabapentin dose had a similar baseline risk of developing altered mental status prior to the initiation of gabapentin, we re-applied the exclusion criteria to our existing cohort on the day that preceded the index date by 180 days. After re-applying exclusions, we followed the retained patients for the same 30-day outcomes. Because there was no plausible reason why the two groups would differ in outcomes prior to the initiation of gabapentin, we reasoned that null associations would enhance assertions that the two groups were similar in their baseline risk for the study outcomes. When we performed this analysis there was no observed association with 30-day risk of the primary study outcome (adjusted odds ratio 1.11, 95% CI 0.91 to 1.35; S6 Table).

### Costing analyses

The adjusted mean costs, are presented in Table 5. A high (vs. low) dose of gabapentin increased the average healthcare cost by $31.22 (average per person 30-day cost with high dose was $64.75, vs. a low dose cost of $33.53). Initiating a high versus low dose of gabapentin was also associated with a higher average per person emergency department cost ($103.70 vs. $90.30), hospitalization cost ($676.07 vs. $598.98), and total healthcare cost ($2,146.38 vs. $1,995.60). A total of 8.2% of the hospital encounters with altered mental status were attributable to initiating a high dose of gabapentin. If the 34,159 patients in our cohort who initiated a high dose of gabapentin had instead started at a low dose of gabapentin, this may have resulted in total healthcare savings of $1,123,281.82 (Table 6).

**Table 5:** Average per person adjusted cost in 30-day follow-up period.

All Patients	Gabapentin	Emergency Visit	Hospitalization	Total Healthcare
High Dose	$64.75	$103.70	$676.07	$2,146.38
Low Dose	$33.53	$90.30	$598.98	$1,995.60
Average Cost Differential	$31.22	$13.41	$77.09	$150.78
P-value	<0.0001	<0.0001	<0.0001	<0.0001

Costing analysis shows average per patient and was adjusted for age, sex, Charlson comorbidity score, index year, trigeminal neuralgia, use of antiepileptics, use of narcotics, and dementia.

**Table 6:** 30-day avoidable healthcare cost of patients hospitalized with altered mental status attributable to high gabapentin dose.

Prevalence of Patients Receiving High Dose Gabapentin	Gabapentin Cost^&^	Emergency Department Cost^&^	Hospitalization Cost^&^	Total Healthcare Cost^&^	30 Day Healthcare Cost Avoidable by Using Low Dose^#^ Gabapentin
31%^$^	$3,697.02	$91,241.52	$994,291.06	$1,123,281.82	---
23.3% (25% reduction)	$2,836.85	$70,012.73	$762,953.47	$861,932.48	$261,349/34
15.5% (50% reduction)	$1,928.03	$47,583.20	$518,530.95	$585,800.69	$537,481.13
7.8% (75% reduction)	$991.42	$24,467.96	$266,636.02	$301,227.08	$822,054.74
0% (100% reduction)*	---	---	---	---	$1,123,281.82

&Attributable cost to high gabapentin dose in patients hospitalized with altered mental status.

#Low dose as described in this study as ≤600mg per day of gabapentin.

$ Actual proportion of patients in our cohort who were initiated on a high dose gabapentin (>600mg per day)

*100% reduction of patients receiving high dose gabapentin = all patients receive low dose.

## Discussion

We conducted this study to characterize the 30-day risk of hospitalization with altered mental status in older adults initiating either a high or low dose of gabapentin in routine outpatient care (using hospitalization with urgent head neuroimaging as a proxy for this outcome, rather than a diagnosis of delirium which could not be reliably assessed in our data sources). Using gabapentin has been shown to provide a statistically significant improvement in quality of life secondary to neuropathic pain reduction when compared to placebo[25]. Compared to a low dose, initiating a high dose of gabapentin was associated with an increased 30-day risk of a hospital encounter for altered mental status, with higher costs to the healthcare system. Fortunately, the risk of being hospitalized with altered mental status after initiating gabapentin remained low, even in patients prescribed a high initial dose of gabapentin.

The efficacy of the lower dosing of gabapentin remains unclear from the current study. Early case reports describe decreased hyperesthesia, decreased episodes of “lancinating” pain, and reduction of burning pain in doses lower than 600mg per day[26]. One randomized clinical trial done in the United Kingdom shows that patients experience improvement of symptoms within one week, which was statistically different from placebo, with doses as low as 900mg per day[25]. Another study showed over 40% of patients achieved moderate or excellent pain relief at 900mg per day[9]. This may mean that doses as low as 600mg per day could provide some benefit, while avoiding risks. The risk of adverse effects would have to be weighed against potential benefits in each patient.

The upper threshold to define our low dose of gabapentin was below the initial recommended dose for pain and several clinical indications. Prior to performing any outcome analysis, we set our dosing thresholds from the average initial prescribed dose, and because these doses could be feasibly assessed in our data sources. It is possible the current standard dose is too high for some older patients, and some hospitalizations may be avoided through the judicious use of gabapentin, use of the lowest dose possible to control pain, and vigilance for early signs of altered mental status. Additionally, the bioavailability of gabapentin is inversely correlated to dosing[27].

In additional analyses we observed an association between high vs. low dose gabapentin and a higher risk of hospitalization for mental status changes in patients both with and without chronic kidney disease. Thus it may be prudent to be cautious about gabapentin dosing in older patients irrespective of their level of kidney function. Gabapentin is eliminated almost entirely by the kidneys, and in our subgroup analyses risks were highest in the presence of chronic kidney disease (Table 4). Our results emphasize the need to consider initiating a low dose of gabapentin particularly in patients with chronic kidney disease.

Our study has several strengths. Ontario offers a large and diverse population in which all residents have universal medical coverage and everyone over 65 years old has prescription drug coverage. This allowed us to study a more generalizable and real practice population, complementing the information available from a Cochrane systematic review of randomized control trials and case reports which assessed different gabapentin dosing[2,28,29]. We used a low dose of gabapentin as a comparator group (versus no gabapentin at all) to reduce concerns about confounding. Finally, our research protocol, cohort, and outcomes were pre-specified, and the results were consistent with our *a priori* hypotheses.

Our study does have some limitations. We did not have details of the indication for gabapentin use or treatment effectiveness, or accurate information on any changes in the dose of gabapentin over 30 days of follow-up from the average daily dose ascertained from the first prescription. Our outcome of hospitalization with altered mental status was ascertained by knowledge of whether a patient was hospitalized and received urgent neuroimaging. With our data sources this was done *a priori* and deemed to be the best way to ascertain the underlying adverse event of interest, and has successfully been used in other studies[30,31]. We reasoned urgent head CT scans done for reasons unrelated to gabapentin dose (i.e. headache) would occur at a similar frequency in the high and low dose groups, and therefore would not impact estimates of difference in risk. Additionally we excluded patients that had a most responsible diagnosis of stroke to avoid this as a potential confounding cause for urgent neuroimaging, although we do not know the indication for the head CT scan. However, a more robust study design would be to follow older patients prospectively after receipt of gabapentin with an independent adjudication of mental status outcomes, recognizing that such a study would be a substantial undertaking with associated costs. Because ours was an observational study we describe associations without definitive proof of causation, and the possibility of residual confounding remains. However, we did not detect a difference in 30-day risk of the primary study outcome between the two groups when the cohort was examined 180 days prior to initiating gabapentin–suggesting the two groups had a similar baseline risk for the study outcomes. Additionally, we cannot assess the effectiveness of the proposed doses based on this current study design. To our knowledge, a randomized control study assessing a dose of 600mg per day vs. higher doses has not been done, and such a study may contribute valuable information to guide dosing and effectiveness in the elderly. Finally, our findings can only be generalized to older adults, as younger patients are often healthier and may not be as susceptible to adverse drug events.

In conclusion, the absolute risk of being hospitalized with altered mental status after the initiation of gabapentin is low. However, when the drug is clinically indicated, some adverse events and associated healthcare costs may be avoided if the lowest dose of gabapentin is used. Future studies should assess if the doses recommended in this article are as effective as higher doses.

## Supporting information

S1 Table STROBE checklist. (DOCX)

S2 Table Listing of CKD codes from previously validated algorithm. (DOCX)

S3 Table Full baseline characteristics. (DOCX)

S4 Table Subgroup analysis for secondary outcome of all-cause mortality in 30 day follow up. (DOCX)

S5 Table Primary analysis using CT head within 2 days of hospital admission. (DOCX)

S6 Table Baseline risk of 30-day outcomes in the 180 days prior to cohort entry. (DOCX)

S1 Fig Cohort selection. (JPG)
