# Supplementary material for: Gabapentin dose and the 30-day risk of altered mental status in older adults: A retrospective population-based study
Source: PLoS One. 2018 Mar 14;13(3):e0193134. doi: 10.1371/journal.pone.0193134 (PMC5851574; doi:10.1371/journal.pone.0193134)
Supplement: S1 Table — (DOCX) [file pone.0193134.s001.docx]

Supplementary Table 1. STROBE checklist

| **STROBE checklist** | | | | |
| --- | --- | --- | --- | --- |
|  | **Item No** | | **Recommendation** | **Reported** |
| **Title and abstract** | 1 | | (a) Indicate the study’s design with a commonly used term in the title or the abstract | Title page |
|  |  |  | (b) Provide in the abstract an informative and balanced summary of what was done and what was found | Abstract |
| **Introduction** | | | |  |
| Background / rationale | 2 | | Explain the scientific background and rationale for the investigation being reported | Introduction |
| Objectives | 3 | | State specific objectives, including any pre-specified hypotheses | Introduction |
| **Methods** | | | |  |
| Study design | 4 | | Present key elements of study design early in the paper | Methods |
| Setting | 5 | | Describe the setting, locations, and relevant dates, including periods of recruitment, exposure, follow-up, and data collection | Methods |
| Participants | 6 | | (a) Give the eligibility criteria, and the sources and methods of selection of participants. Describe methods of follow-up | Methods |
|  |  |  | (b) For matched studies, give matching criteria and number of exposed and unexposed | Methods |
| Variables | 7 | | Clearly define all outcomes, exposures, predictors, potential confounders, and effect modifiers. Give diagnostic criteria, if applicable | Methods |
| Data sources/ measurement | 8 | | For each variable of interest, give sources of data and details of methods of assessment (measurement). Describe comparability of assessment methods if there is more than one group | Methods |
| Bias | 9 | | Describe any efforts to address potential sources of bias | Methods |
| Study size | 10 | | Explain how the study size was arrived at | Methods |
| Quantitative variables | 11 | | Explain how quantitative variables were handled in the analyses. If applicable, describe which groupings were chosen and why | Methods |
| Statistical methods | 12 | | (a) Describe all statistical methods, including those used to control for confounding | Methods |
|  |  |  | (b) Describe any methods used to examine subgroups and interactions | Methods |
|  |  |  | (c) Explain how missing data were addressed | (N/A) |
|  |  |  | (d) If applicable, explain how loss to follow-up was addressed | Methods |
|  |  |  | (e) Describe any sensitivity analyses | Methods |
| **Results** | | | |  |
| Participants | 13 | (a) Report numbers of individuals at each stage of study—e.g. numbers potentially eligible, examined for eligibility, confirmed eligible, included in the study, completing follow-up, and analyzed | | Results  Appendix C |
|  |  | (b) Give reasons for non-participation at each stage | | N/A |
|  |  | (c) Consider use of a flow diagram | | Appendix C |
| Descriptive data | 14 | (a) Give characteristics of study participants (e.g. demographic, clinical, social) and information on exposures and potential confounders | | Results  Table 2 |
|  |  | (b) Indicate number of participants with missing data for each variable of interest | | N/A |
|  |  | (c) Summarize follow-up time (e.g. average and total amount) | | Results |
| Outcome data | 15 | Report numbers of outcome events or summary measures over time | | Results  Table 3,4 |
| Main results | 16 | (a) Give unadjusted estimates and, if applicable, confounder-adjusted estimates and their precision (e.g. 95% confidence interval). Make clear which confounders were adjusted for and why they were included | | Results  Table 3,4 |
|  |  | (b) Report category boundaries when continuous variables were categorized | | Results  Table 3,4 |
|  |  | (c) If relevant, consider translating estimates of relative risk into absolute risk for a meaningful time period | | N/A |
| Other analyses | 17 | Report other analyses done—e.g. analyses of subgroups and interactions, and sensitivity analyses | | Results  Table 4,5,6 |
| **Discussion** | | | |  |
| Key results | 18 | Summarize key results with reference to study objectives | | Discussion |
| Limitations | 19 | Discuss limitations of the study, taking into account sources of potential bias or imprecision. Discuss both direction and magnitude of any potential bias | | Discussion |
| Interpretation | 20 | Give a cautious overall interpretation of results considering objectives, limitations, multiplicity of analyses, results from similar studies, and other relevant evidence | | Discussion |
| Generaliza-bility | 21 | Discuss the generalizability (external validity) of the study results | | Discussion |
| **Other information** | | | |  |
| Funding | 22 | Give the source of funding and the role of the funders for the present study and, if applicable, for the original study on which the present article is based | | Disclosure |
