# Supplementary material for: Gabapentin dose and the 30-day risk of altered mental status in older adults: A retrospective population-based study
Source: PLoS One. 2018 Mar 14;13(3):e0193134. doi: 10.1371/journal.pone.0193134 (PMC5851574; doi:10.1371/journal.pone.0193134)
Supplement: S2 Table — (DOCX) [file pone.0193134.s002.docx]

Supplementary Table 2. Listing of CKD codes from previously validated algorithm.

| **ICD 10 Code** | **Description** |
| --- | --- |
| E102 | Type 1 diabetes mellitus with incipient diabetic nephropathy adequately or inadequately controlled with insulin, diet, oral agents |
| E112 | Type 2 diabetes mellitus with incipient diabetic nephropathy adequately or inadequately controlled with insulin, diet, oral agents |
| E132 | Other specified diabetes mellitus with incipient diabetic nephropathy adequately or inadequately controlled with insulin, diet, oral agents |
| E142 | Unspecified diabetes mellitus with incipient diabetic nephropathy adequately or inadequately controlled with insulin, diet, oral agents |
| I12 | Hypertensive renal disease |
| I13 | Hypertensive renal and heart disease |
| N08 | Glomerular disorders in diseases classified elsewhere |
| N18 | Chronic renal failure |
| N19 | Unspecified renal failure |
| **OHIP Diagnosis Code** |  |
| 403 | Hypertensive renal disease |
| 585 | Chronic renal failure, uremia |
