# Supplementary material for: Gabapentin dose and the 30-day risk of altered mental status in older adults: A retrospective population-based study
Source: PLoS One. 2018 Mar 14;13(3):e0193134. doi: 10.1371/journal.pone.0193134 (PMC5851574; doi:10.1371/journal.pone.0193134)
Supplement: S3 Table — (DOCX) [file pone.0193134.s003.docx]

Supplementary Table 3. Full Baseline Characteristics Table

|  | High Dose^¶^  n = 34,159 | Low Dose^£^  n = 76,025 | Standardized Difference (%)^*^ |
| --- | --- | --- | --- |
| Age, mean (SD) | 74.4 (6.5) | 76.3 (7.2) | 28% |
| Women | 19,508 (57.1) | 48,906 (64.3) | 15% |
| Year of cohort entry |  |  |  |
| 2002-2005 | 4,067 (11.9) | 4,272 (5.6) | 22% |
| 2006-2009 | 6,187 (18.1) | 8,219 (10.8) | 21% |
| 2010-2013 | 19,563 (57.3) | 48,743 (64.1) | 14% |
| 2014 | 4,342 (12.7) | 14,791 (19.5) | 19% |
| Rural | 5,418 (15.9) | 10,464 (13.8) | 6% |
| Income quintile |  |  |  |
| Missing | 247 (0.3) | 133 (0.4) | 2% |
| 1 (lowest) | 7,049 (20.6) | 16,120 (21.2) | 1% |
| 2 | 6,997 (20.5) | 16,009 (21.1) | 1% |
| 3 | 6,655 (19.5) | 15,179 (20.0) | 1% |
| 4 | 6,743 (19.7) | 14,621 (19.2) | 1% |
| 5 (highest) | 6,582 (19.3) | 13,849 (18.2) | 3% |
| Number of hospitalizations in the prior one year, mean (SD) | 0.74 (1.24) | 0.82 (1.32) | 6% |
| At home physician services | 4,781 (6.3) | 1,817 (5.3) | 4% |
| *Comorbidities in 5 years prior* |  |  |  |
| Acute urinary retention | 962 (2.8) | 1,943 (2.6) | 1% |
| Alcoholism | 773 (2.3) | 1,398 (1.8) | 4% |
| Alzheimer’s disease | 62 (0.2) | 238 (0.3) | 2% |
| Angina | 7,506 (22.0) | 16,296 (21.4) | 1% |
| Atrial fibrillation/flutter | 2,414 (7.1) | 5,969 (7.9) | 3% |
| Bipolar disorder | 1,013 (3.0) | 2,308 (3.0) | 0% |
| Cardiovascular disease^#^ | 10,056 (29.4) | 22,693 (29.8) | 1% |
| Chronic liver disease | 1,599 (4.7) | 3,328 (4.4) | 1% |
| Chronic lung disease | 11,376 (33.3) | 24,885 (32.7) | 1% |
| Chronic obstructive pulmonary disease | 2,105 (6.2) | 4,460 (5.9) | 1% |
| Dementia | 3,153 (9.2) | 8,866 (11.7) | 8% |
| Depression/anxiety | 4,339 (12.7) | 9,575 (12.6) | 0% |
| Diabetes Mellitus | 8,650 (25.3) | 20,008 (26.3) | 2% |
| Diabetic Neuropathy | 507 (1.5) | 747 (1.0) | 5% |
| Congestive heart failure | 4,663 (13.7) | 11,683 (15.4) | 5% |
| Hypothyroidism | 730 (2.1) | 1,710 (2.2) | 1% |
| Migraine | 2,181 (6.4) | 4,370 (5.7) | 3% |
| Neuropathic Pain | 1,778 (5.2) | 2,675 (3.5) | 8 % |
| Parkinson’s disease | 1,004 (2.9) | 2,241 (2.9) | 0% |
| Peripheral vascular disease | 1,068 (3.1) | 1,871 (2.5) | 4% |
| Prostatic hyperplasia | 5,125 (15.0) | 9,783 (12.9) | 6% |
| Prostatitis | 1,628 (4.8) | 2,925 (3.8) | 5% |
| Schizophrenia and other Psychotic Disorders | 923 (2.7) | 2,239 (2.9) | 1% |
| Seizure disorder | 528 (1.5) | 728 (1.0) | 5% |
| Sepsis | 576 (1.7) | 1,109 (1.5) | 2% |
| Stroke | 1,227 (3.6) | 2,602 (3.4) | 1% |
| Trigeminal Neuralgia | 2,249 (6.6) | 3,205 (4.2) | 11% |
| *Tests/Procedures in the one year prior* |  |  |  |
| Carotid ultrasound | 2,401 (7.0) | 5,252 (6.9) | 0% |
| Carotid endarterectomy | 29 (0.1) | 58 (0.1) | 0% |
| Cardiac catheterization | 771 (2.3) | 1,561 (2.1) | 1% |
| Echocardiography | 7,567 (22.2) | 18,122 (23.8) | 4% |
| Holter monitoring | 2,612 (7.6) | 6,332 (8.3) | 3% |
| Cardiac stress test | 5,003 (14.6) | 10,934 (14.4) | 1% |
| Colorectal cancer screening | 3,251 (9.5) | 6,073 (8.0) | 5% |
| Cervical cancer screening | 1,357 (4.0) | 2,860 (3.8) | 1% |
| Prostate specific antigen test | 1,667 (4.9) | 3,447 (4.5) | 2% |
| Mammography | 3,729 (10.9) | 8,910 (11.7) | 3% |
| Flu shot | 18,068 (52.9) | 40,724 (53.6) | 1% |
| Pneumococcal vaccine | 226 (0.7) | 678 (0.9) | 2% |
| Bone mineral density test | 3,653 (10.7) | 8,550 (11.2) | 2% |
| Hearing test | 1,835 (5.4) | 4,361 (5.7) | 1% |
| Cystoscopy | 2,019 (5.9) | 4,147 (5.5) | 2% |
| Transurethral resection of prostate | 194 (0.6) | 370 (0.5) | 1% |
| Cataract surgery | 1,686 (4.9) | 3,705 (4.9) | 0% |
| CT head | 5,628 (16.5) | 12,067 (15.9) | 2% |
| CT neck | 785 (2.3) | 1,424 (1.9) | 3% |
| CT thorax | 4,746 (13.9) | 9,291 (12.2) | 5% |
| CT abdomen | 6,017 (17.6) | 12,113 (15.9) | 5% |
| CT pelvis | 5,610 (16.4) | 11,363 (14.9) | 4% |
| CT spine | 2,739 (8.0) | 5,024 (6.6) | 5% |
| CT extremity | 699 (2.0) | 1,403 (1.8) | 1% |
| Chest x-ray | 15,700 (46.0) | 34,783 (45.8) | 0% |
| Pulmonary function test | 4,237 (12.4) | 8,779 (11.5) | 3% |
| Electroencephalogram | 583 (1.7) | 871 (1.1) | 5% |
| Heart Valve Replacement | 62 (0.2) | 134 (0.2) | 0% |
| Cholesterol test | 18 (0.1) | 64 (0.1) | 0% |
| Interventional Pain Nerve Block | 1,819 (5.3) | 3,577 (4.7) | 3% |
| *Prescribing Physician Characteristics^**^* |  |  |  |
| Time since graduation, mean (SD) | 26.2 (11.7) | 25.7 (11.7) | 4% |
| *Specialty* |  |  |  |
| GP | 22,346 (65.4) | 53,403 (70.2) | 10% |
| Anesthesiologist | 441 (1.3) | 1,102 (1.4) | 1% |
| Nephrology | 25 (0.1) | 336 (0.4) | 6% |
| Cardiology | 79 (0.2) | 183 (0.2) | 0% |
| Neurology | 2,510 (7.3) | 3,184 (4.2) | 13% |
| Physical Medicine and Rehab | 400 (1.2) | 805 (1.1) | 1% |
| Missing | 9,145 (12.0) | 5,037 (14.7) | 8% |
| Other | 3,321 (9.7) | 7,867 (10.3) | 2% |
| Rural practice | 3,355 (9.8) | 6,725 (8.8) | 3% |
| *Medications in 180 days prior* |  |  |  |
| ACE-inhibitors | 10,712 (31.4) | 22,834 (30.0) | 3% |
| ARBs | 5,886 (17.2) | 15,186 (20.0) | 7% |
| Amiodarone/digoxin | 1,465 (4.3) | 3,468 (4.6) | 1% |
| Anti-depressants | 11,984 (35.1) | 24,114 (31.7) | 7% |
| Anti-epileptics | 2,418 (7.1) | 3,188 (4.2) | 13% |
| Antipsychotics | 1,485 (4.3) | 3,839 (5.0) | 3% |
| Beta-blockers | 9,831 (28.8) | 23,096 (30.4) | 4% |
| Calcium channel blockers | 10,102 (29.6) | 24,239 (31.9) | 5% |
| Fibrates | 867 (2.5) | 1,515 (2.0) | 3% |
| Diuretics | 11,172 (32.7) | 25,337 (33.3) | 1% |
| Diuretic combination drugs | 3,639 (10.7) | 8,666 (11.4) | 2% |
| Histamine-2 receptor antagonist | 2,546 (7.5) | 5,306 (7.0) | 2% |
| Statins | 16,982 (49.7) | 39,603 (52.1) | 5% |
| Benzodiazepines | 7,732 (22.6) | 16,783 (22.1) | 1% |
| Cholinesterase inhibitors | 709 (2.1) | 2,243 (3.0) | 6% |
| Migraine therapies | 15 (0.03) | 20 (0.04) | 6% |
| Narcotics and Narcotic Antagonists | 18,516 (54.2) | 36,136 (47.5) | 13% |
| NSAIDs including ASA | 12,175 (35.6) | 26,129 (34.4) | 3% |
| Overactive Bladder Medications | 1,678 (4.9) | 3,878 (5.1) | 1% |
| Abbreviations: SD, standard deviation; CT, computed tomography; GP, general practitioner; CMG, Canadian Medical Graduate; ACE, angiotensin converting enzyme; ARB, angiotensin receptor blocker; NSAIDs, nonsteroidal anti-inflammatory drug; ASA, acetylsalicylic acid  ^¶^High dose of gabapentin defined as >600 mg/day  ^£^Low dose of gabapentin defined as ≤600 mg/day  *Standardized differences are less sensitive to sample size than traditional hypothesis tests. They provide a measure of the difference between groups divided by the pooled standard deviation; a value greater than 10% is interpreted as a meaningful difference between the groups  #Coronary artery disease incorporates coronary artery revascularization as well as myocardial infarction but does not include angina  **Physicians may be presented more than once if they had written prescriptions for more than 1 patient present in our cohort. | | | |
