# Supplementary material for: Gabapentin dose and the 30-day risk of altered mental status in older adults: A retrospective population-based study
Source: PLoS One. 2018 Mar 14;13(3):e0193134. doi: 10.1371/journal.pone.0193134 (PMC5851574; doi:10.1371/journal.pone.0193134)
Supplement: S4 Table — (DOCX) [file pone.0193134.s004.docx]

Supplementary Table 4. Subgroup analysis for secondary outcome of all-cause mortality in 30 day follow up

| **CKD Status** | **Dose^$^** | **Number of patients** | **Number of events^€^, n (%)** | **Relative Risk (95% CI)** | | **Adjusted p- value** | **Adjusted interaction p-value** |
| --- | --- | --- | --- | --- | --- | --- | --- |
|  |  |  |  | Unadjusted | Adjusted |  |  |
| **Chronic kidney disease^*^** | Low Dose | 8,345 | 186 (2.23) | 0.99 (0.74 – 1.31) | 1.04 (0.78 – 1.40) | 0.78 | 0.97 |
|  | High Dose | 2,955 | 65 (2.20) |  |  |  |  |
| **No chronic kidney disease^*^** | Low Dose | 67,680 | 697 (1.03) | 1.12 (0.99 – 1.28) | 0.99 (0.86 – 1.13) | 0.84 |  |
|  | High Dose | 31,204 | 361 (1.16) |  |  |  |  |
| **eGFR <45^#^** | Low Dose | 2,729 | 44 (1.61) | 1.32 (0.76 – 2.30) | 1.48 (0.84 – 2.62) | 0.18 | 0.35 |
|  | High Dose | 848 | 18 (2.12) |  |  |  |  |
| **eGFR ≥45^#^** | Low Dose | 15,454 | 131 (0.85) | 1.06 (0.78 – 1.45) | 0.96 (0.70 – 1.32) | 0.81 |  |
|  | High Dose | 6,653 | 60 (0.90) |  |  |  |  |
| Abbreviations: CKD, chronic kidney disease; eGFR, estimated glomerular filtration rate;  $ Low dose defined as ≤600mg per day of gabapentin. High dose defined as >600mg per day of gabapentin.  € Events are defined by all-cause mortality  * Chronic kidney disease as defined by presence of at least one administrative database code from a previously validated algorithm. Listing of codes is presented in Appendix B  # eGFR is based on subgroup analysis of patients that have a recent serum creatinine laboratory value from Gamma-Dynacare or Cerner.  Patients prescribed the low gabapentin dose served as the referent group. | | | | | | | |
