# Supplementary material for: Gabapentin dose and the 30-day risk of altered mental status in older adults: A retrospective population-based study
Source: PLoS One. 2018 Mar 14;13(3):e0193134. doi: 10.1371/journal.pone.0193134 (PMC5851574; doi:10.1371/journal.pone.0193134)
Supplement: S5 Table — (DOCX) [file pone.0193134.s005.docx]

Supplementary Table 5. Primary analysis using CT head within 2 days of hospital admission.

|  | **Number of events, n (%)** | | **Relative Risk (Unadjusted)**  **(95% CI)** | **Relative Risk (Adjusted)**^¥^  **(95% CI)** |
| --- | --- | --- | --- | --- |
|  | **High Dose Group**^£^  **N = 34,159** | **Low Dose Group**^£^  **N = 76,025** |  |  |
| **Hospitalization with altered mental status*** | 427 (1.25) | 798 (1.05) | 1.19  (1.06 – 1.34) | 1.29  (1.14 – 1.46) |
| ^£^Low and high dose group as per previous allocation into groups  ^¥^Adjusted for 8 covariates (see Methods)  * Altered mental status as defined by receipt of urgent head CT scan in the absence of diagnosis of stroke within the first 2 days of hospital admission as diagnosed by hospital administrative codes. Patients prescribed the low gabapentin dose served as the referent group.  Abbreviations: CI, confidence interval | | | | |
