# Supplementary material for: Gabapentin dose and the 30-day risk of altered mental status in older adults: A retrospective population-based study
Source: PLoS One. 2018 Mar 14;13(3):e0193134. doi: 10.1371/journal.pone.0193134 (PMC5851574; doi:10.1371/journal.pone.0193134)
Supplement: S6 Table — (DOCX) [file pone.0193134.s006.docx]

Supplementary Table 6. Additional Analysis - Baseline risk of 30-day outcomes in the 180 days prior to cohort entry.

|  | **Number of events, n (%)** | | **Relative Risk (Unadjusted)**  **(95% CI)** | **Relative Risk (Adjusted)**^¥^  **(95% CI)** |
| --- | --- | --- | --- | --- |
|  | **High Dose Group**^£^  **N = 31,078** | **Low Dose Group**^£^  **N = 71,618** |  |  |
| **Hospitalization with altered mental status*** | 151 (0.49) | 319 (0.45) | 1.09  (0.90 – 1.33) | 1.11  (0.91 – 1.35) |
| Abbreviations: CI, confidence interval  ^£^Low and high dose group as per previous allocation into groups  ^¥^Adjusted for 8 covariates (see Methods)  * Altered mental status as defined by receipt of urgent head CT scan in the absence of diagnosis of stroke within the first 5 days of hospital admission as diagnosed by hospital administrative codes.  Patients prescribed the low gabapentin dose served as the referent group. | | | | |
